# Bayesian Architecture for Predictive Monitoring of Unbalance Faults in a Turbine Rotor–Bearing System

**DOI:** 10.3390/s24248123

**Published:** 2024-12-19

**Authors:** Banalata Bera, Shyh-Chin Huang, Po Ting Lin, Yu-Jen Chiu, Jin-Wei Liang

**Affiliations:** 1 Department of Mechanical Engineering, National Taiwan University of Science and Technology, Taipei City 10607, Taiwan; d10803807@mail.ntust.edu.tw (B.B.); potinglin@mail.ntust.edu.tw (P.T.L.); 2 Department of Mechanical Engineering, Ming Chi University of Technology, New Taipei City 24301, Taiwan; yjchiu@mail.mcut.edu.tw (Y.-J.C.); liangj@mail.mcut.edu.tw (J.-W.L.)

**Keywords:** Bayesian model updating, MCMC, model-based diagnosis, unbalance prognosis and monitoring, uncertainty analysis

## Abstract

Unbalance faults are among the common causes of interruptions and unexpected failures in rotary systems. Therefore, monitoring unbalance faults is essential for predictive maintenance. While conventional time-invariant mathematical models can assess the impact of these faults, they often rely on proper assumptions of system factors like bearing stiffness and damping characteristics. In reality, continuous high-speed operation and environmental factors like load variations cause these parameters to change. This work presents a novel architecture for unbalance fault monitoring and prognosis, in which the bearing parameters are treated as variables that change with operating conditions. This enables the development of a more reliable mathematical model for continuous monitoring and prognosis of unbalance faults in rotor systems. This Bayesian inference framework uses Markov Chain Monte Carlo (MCMC) sampling to identify dynamic bearing parameters. Specifically, the Metropolis algorithm is employed to systematically evaluate the range of acceptable parameter values within the framework. A novel dual-MCMC loops explore and assess the parameter space, resulting in more accurate and reliable bearing parameter estimations. These updated parameters improve the demonstrated turbine rotor–bearing system’s unbalance assessment up to 74.48% of the residual error compared to models with fixed parameters. This validates the Bayesian framework for predictive monitoring and maintenance-oriented solutions.

## 1. Introduction

Systems with rotating components form the core of almost all engineering systems, such as power generation, aerospace, automobiles, etc., making them an indispensable element for the maintenance of overall efficiency and reliability of the system. Nevertheless, they are also prone to severe failures due to continuous operations at high speeds and heavy loads, which may cause unwanted shutdowns or even catastrophic accidents in any industry. Thus, ensuring their safe operation and effective maintenance has been a subject of concern for many decades. Lee et al. [1] provided an in-depth analysis of a systematic method for developing Prognostics and Health Management (PHM) for rotating systems. Thia methodology facilitates early detections of potential faults that may occur and help in taking preventive maintenance-based actions beforehand. Out of various faults that commonly occur in a rotor system, such as unbalance, misalignment, shaft bow, rub, etc., unbalance has been one of the major contributing faults, which also adds extra vibrations, resulting in various secondary faults in the system if not maintained in time. Unbalance faults occur due to the rotating systems’ mass center not being perfectly aligned with its center of rotation. This alignment becomes altered due to factors such as continuous operation at high speeds and excessive loads within the machinery, making this type of fault difficult to completely get rid of. Thus, diagnosing this type of fault becomes extremely critical in view of regular maintenance. Walker et al. [2] inferred that even if the existence of an unbalance fault is inherent in every system, a lot of challenges are faced in accurately determining its severity in a system, due to the lower availability of failure data from real-life operating systems.

Thus, in regard to the above, condition-based monitoring (CBM) [3,4,5] has been utilized to determine the current state of a system by identifying the different fault features present in the system and taking prompt preventive decisions based on that. Regardless of this fact, earlier identification of faults has garnered the undivided attention of researchers owing to its significance in reduction in downtime and avoiding safety-associated issues in the industry, it still poses challenges in terms of preciseness. For years, many advanced signal processing methods have been developed for accurate and efficient monitoring of rotating machinery in the industry. Within CBM, vibration-based condition monitoring [6,7,8] has been one of the predominant approaches in solving and analyzing the faults of rotor systems, since vibration can capture the inherent nature of the system and can identify any changes to it. Furthermore, the diagnostic approaches to faults of rotating systems are generally categorized into model-based, data-driven, also known as signal-based, or knowledge-based architectures, described more in studies [2,9,10,11,12]; there is another recent procedure, labeled as a hybrid approach, which combines elements from these different methodologies. However, hybrid approaches [13,14,15] have very few studies so far, due to them adding more complications to the overall diagnosis procedure. Data-driven methods rely exclusively on signal processing algorithms and data from different sensors for analysis and trend development, yet they do not consider the general physics of the system and hence sometimes fail to comprehend the mechanical principles governing a system. Also, in the absence of adequate data, data-driven fault diagnosis fails to provide accurate and reliable diagnosis.

Model-based methods (MBMs) [16,17,18,19,20], on the other hand, provides more precise and consistent assessments for the identification of faults in rotary systems. Though MBMs are good for accuracy and understanding the nature of the system, they can be difficult to build up for complex real-life industrial rotor systems. These methods develop system formulations by utilizing the fundamental principles of physics and generally depend on several parameters that control the behavior of the system. While some rotor system characteristics, such as mass and size, etc., may be easily determined by simple calculations, others, such as bearing parameters, provide difficulties since they are not directly measurable. The majority of model-based fault identification algorithms presuppose the availability of established bearing parameters. Nonetheless, it is considered essential to thoroughly assess the bearing properties in a rotor–bearing system for effective unbalance monitoring.

Bearing parameters can be determined by utilizing optimization algorithms that use the measured response obtained from either test rigs or actual operational machines. Martin [21] performed an experimental investigation for the optimization of a hydrostatic bearing parameters and compared his results with established empirical standards. By using advanced measurement techniques involving displacement-based coefficients, the bearing stiffness parameters estimated came out to be significantly lesser than the empirical ones. Hence, it was cautioned not to rely exclusively on theoretical or empirical methods to determine these coefficients, since they are highly susceptible to factors such as film thickness, alignment, and assembly tolerance. These factors might change in each assembly, hence illustrating the importance of advanced calculations based on different factors. In another study, Mao et al. [19] developed an analytical model of a rotor–bearing system using the transfer matrix method, wherein they determined the bearing parameters by minimizing the error in unbalance response between the experimental findings obtained from a test rig and the corresponding computational results. In a hydrodynamic bearing, the dynamic characteristics of the bearing depend on factors such as speed, eccentric ratio, and other variables affecting the system. Estimating these values often involves complex and detailed calculations. Mutra and Srinivas [22] utilized a Finite Element Method (FEM) model to analyze a rotor–bearing system. They employed an error-based formulation, specifically focusing on the response amplitudes at bearing nodes. Their objective was to determine the speed-dependent stiffness and damping parameters of a hydrodynamic bearing. To achieve this, they employed a modified particle swarm optimization algorithm with mutation to search for the optimal solution. The methodology’s robustness was verified by injecting varying degrees of noise into the observed reference signals.

Research efforts have recently increased in the study of the complexities of parameter identification methods for rotor systems, specifically in simultaneously detecting rotor unbalances and bearing parameters. For example, Tiwari and Chakravarthy [23] presented a groundbreaking method in which they used data from impulsive response and rig rundown tests of a rotor–bearing system to estimate residual unbalance and bearing parameters concurrently. Their method was validated by checking the identified unbalance masses with the residual masses taken and they matched well in a dynamically balanced rotor–bearing test rig, proving the significance of their method. Generally, all of these studies mostly involve idealized states or properly maintained experimental setups or test rigs; therefore, the bearing characteristics do not change much. However, in real-life scenarios the bearing characteristics of complex structures may vary during operation due to various factors in operation. According to a previous study conducted by the authors of this research [24], it was shown that the bearing coefficients underwent considerable changes during operation, resulting in substantial inaccuracies when detecting unbalance, thus imposing a need for more advance computations in this regard.

In general, there are two primary approaches to parameter estimation in mathematical optimization: deterministic and stochastic [25,26,27]. Deterministic approaches presume complete knowledge of all parameters, neglecting uncertainty and perhaps missing useful alternatives. Although these methods provide dependable and faster solutions owing to their simplicity, their inflexibility may result in inferior performance under unforeseeable circumstances. Stochastic or probabilistic optimization explicitly incorporates uncertainty by considering parameters as random variables with known or unknown probability distributions. These strategies succeed in domains marked by significant unpredictability, such as risk management, machine learning, and complex systems analysis, by concentrating on average or anticipated favorable results. As a potent instrument for quantifying uncertainty, Bayesian inference has found interest in domains such as system identification, fault detection, and model updating of rotating systems in recent years [25,28]. Garoli et al. [29] demonstrated a stochastic methodology for identification of unbalance and bearing wear fault parameters using Bayesian inference, which was evaluated with the help of polynomial chaos expansion. Their research also showcased a comparison between stochastic and deterministic methodologies in the presence of measurement uncertainty and found that both methodologies are promising; however, the stochastic method can explain the presence of uncertainty in a situation better.

Engineering systems inherently have uncertainties, which should be measured in order to provide more accurate models. Most of the research related to uncertainties generally deals with uncertainty quantification (UQ) [30,31], which examines the effect of the presence of uncertainties on the outputs of a model. However, it is of equal importance to reevaluate or re-update some of the model parameters that are known to have a higher degree of inaccuracy, to achieve a more reliable system model as these parameters change their nature with time or due to the unexpected occurrence of any event during operation. In this regard, Bayesian inference [32,33,34,35] is being utilized to identify or update the probability distributions of variables of interest in the field of rotor dynamics. This can be seen in research: Herp et al. [36] contributed a statistical approach to predict wind turbine failure states in bearings with the help of a Bayesian inference-based methodology for wind turbines. They predicted bearing failure on average 33 days ahead of time using bearing temperature residuals and Gaussian processes. Bayesian inference was also utilized to evaluate the acoustic mode identification problem in the presence of uncertainty in a study by Roncen et al. [37].

The inference problem generally involves the presence of solving for the integral sum, which can be complex in nature and difficult to resolve and is therefore replaced by sampling techniques for solutions. The most straightforward and simplistic sampling technique for the Bayesian approach is the use of Monte Carlo simulations (MCSs) [38,39]. MCS encompasses a huge number of stochastic simulation techniques that can be employed to solve inference-based problems. It takes a substantial number of potential candidates and generates iteratively the best choice of candidates via probability-based calculations. Markov Chain Monte Carlo (MCMC) methods enhance conventional MCS techniques by utilizing Markov chains, facilitating sampling from complex probability distributions where direct sampling is unfeasible. The Metropolis [40] algorithm is a crucial MCMC approach that enhances computational efficiency in high-dimensional environments by repeatedly accepting or rejecting sample candidates according to probability ratios.

Bayesian-based model updating makes use of the inverse UQ technique to update the model parameters [41]. By incorporating this analysis into the diagnostic process, engineers can have greater confidence in the results and make more informed decisions regarding maintenance and repairs. Bayesian inference improves this process by offering a systematic framework for updating model predictions based on prior knowledge and observational data. The use of Bayesian-based inference analysis allows for a more thorough understanding of the potential variability in the bearing parameters, leading to a more reliable and accurate model. Hosseinpour et al. [42] conducted a study where they introduced a very effective and dependable Bayesian approach for prognostics and also calculated the remaining usable life (RUL) of a battery. By continuously refining and updating the model based on this analysis, engineers can ensure that the system operates with optimal efficiency and reliability. In another study by He et al. [43], an adaptive Gaussian process model-based updating method was developed for a rotor system model for the prediction of dynamic responses by using unbalance responses. They utilized a multiple sensor data fusion strategy to develop an objective function for their model updating approach. Their methodology showed a good agreement between the updated and measured model’s modal frequency.

MCMC and Bayesian inference methodologies have proven effective in several domains characterized by significant uncertainty [44,45]. These approaches are especially beneficial for integrating existing knowledge with new input, rendering them suitable for systems characterized by considerable unpredictability. But even with their success in a variety of applications, there is a conspicuous lack of research focused on using these approaches to precisely identify rotor system parameters such as bearing parameters in the context of rotor dynamics. Considerations like wear and tear, operating circumstances, manufacturing variability, and environmental considerations can all lead to uncertainty in bearing specifications. Therefore, to guarantee the best possible performance, dependability, and longevity of rotating machinery, this uncertainty must be effectively addressed. MCMC techniques such as random-walk Metropolis (RWM) may face convergence challenges if the proposal distribution is inadequately selected, leading to slower convergence and unreliable posterior density estimations. Some adaptive MCMC methods have been established in recent years to solve this issue, providing support for their respective problems.

Sun et al. [46] used hydrodynamic bearings to study the stability of rotor–bearing systems affected by misalignment faults and bearing parameter uncertainties. They utilized generalized polynomial chaos (gPC) expansion in their study to quantify the parameter-based uncertainty. In another study by Cavalini et al. [47], they examined the effect of uncertainties on the dynamic behavior of a flexible rotor system supported by cylindrical fluid-film bearings. They employed a fuzzy logic-based analysis to handle stochastic uncertainty and compared their results with experimental data. Furthermore, Taherkhani and Ahmadian [48] utilized a delayed rejection adaptive Metropolis algorithm (DRAM)-based methodology for the stochastic model parameter updating of a complex turbo compressor rotor system. The dynamic coefficients of a tilted pad bearing were observed to change notably in the presence of varying clearances and were updated using their proposed methodology. They evaluated the variation in bearing parameters as a function of the change in rotation speed. Their FEM model system undergoes repeated modifications and reevaluations utilizing their technique, resulting in substantial computing costs throughout the model update process. Despite being a groundbreaking study in this field, it mostly focuses on updating an offline model using pre-collected data. Building a real-time system model where the system parameters can vary during operation and continuously update the model is still a challenging area and remains unexplored. Real-time model updating could enhance overall model robustness and help in the accurate monitoring of fault parameters in rotary systems in the presence of uncertainty.

The use of prognostics to resolve the problem of unbalance faults in rotary systems has posed considerable hurdles, resulting in a shortage of studies in this field to date [1,49]. By using several sensors and vibration-based analysis, predictions can be made about the overall unbalance faults imposed on a system. The use of forecasting methodologies may enable us to examine the collected data, facilitating the anticipation of future discrepancies. This may provide us with substantial time- and cost-saving decisions for the efficient functioning of the overall system. Forecasting methods are essential for anticipating upcoming system characteristics by analyzing trends in historical data. Consequently, classical or statistical approaches, such as exponential smoothing (ES) and auto-regressive integrated moving average (ARIMA) [50], have been used for forecasting works for many years. Recently, there has been an increase in the integration of machine learning (ML) into the field of prognosis to comprehend the intricate structure of systems and to enhance the accuracy of long-term forecasts. Among these methodologies, long short-term memory (LSTM) [51] is an emerging machine learning technique that is gaining traction in time-series forecasting due to its capability to learn long-term relationships in complicated data systems and regression problems.

In our previous research [20,24], an assumed mode method (AMM) was utilized to build a mathematical model derived from physics for an industrial turbine rotor–bearing system for the identification of rotor unbalance and its real-time monitoring, as well as the prediction of future trends in unbalance faults for the development of maintenance-based actions. This model was validated using an FEM model and its closeness with the actual system was verified. The research also found and discussed the real-life problem where the parameter values can change suddenly due to unknown causes during operation. The problem was solved by manually re-identifying the parameters using PSO in [24], whenever large errors in the system model were discovered for a span of three consecutive days. Nevertheless, it remains a challenge to accurately evaluate parameters like bearing parameters, as they have a varying nature and considering them as a constant quantity, as in our earlier study, might not be good for a robust system model. In this research, to contribute further to rotor fault diagnosis and particularly the model updating literature, we have used our turbine rotor–bearing system mathematical model and implemented parameter updating using Bayesian inference to update the mathematical model to be a more accurate and reliable representation of the system under operation.

Thus, this paper presents an extension to earlier research [24]; here, we have utilized a Bayesian-based dual-loop MCMC model updating framework to identify the bearing parameters in the presence of uncertainty and update the mathematical model in real time. At the same time, we simultaneously identify the unbalance parameters. The proposed parameter estimation architecture involves two different MCMC loops based on different criteria for a more extensive and elaborate search of the parameter space. An acceptable sample of parameters from a range of generated samples are chosen to evaluate the real-time unbalance for continuous monitoring. Numerical evaluations involve a comparison of responses from the updated mathematical model and the sensor data from the real-life industrial turbine rotor–bearing system in operation using the residual error. A comparison of the results of our proposed and earlier methodologies has also been described in this paper to demonstrate the novelty and superiority of this method. This research illustrates how it can help in reducing the uncertainty in parameters as the parameter values evolve with time.

The rest of this paper is organized as follows: a detailed description about Bayesian-based model updating is presented and introduced in Section 2. Section 3 describes our mathematical model system and Section 4 briefly explains our proposed parameter estimation methodology. Further, Section 5 discusses in more detail the results and efficacy of our proposed methodology as well as unbalance prognosis. Finally, Section 6 concludes the outcomes derived from this research.

## 2. Bayesian Model Updating

Bayesian model updating is a sophisticated methodology which makes use of Bayesian inference to extract information from prior knowledge and new observation data to improve the estimates of uncertain parameters of the model. It is based on Bayes’ theorem, which takes a prior distribution or initial information of the parameters to be evaluated as probability distributions and evaluates a posterior distribution based on a calculated likelihood function of the observed values.

Assuming M is a model that represents the relationship between the parameters of interest θ and measured response Y, then the updated posterior probability density functions (PDFs) of the parameters are given by Bayes’ theorem as

(1) $$P(\theta \;|\;Y,M)=\frac{P(\left.Y\right|\theta ,M)\times P(\left.\theta \right|M)}{P(\left.Y\right|M)}$$

where $P(\left.\theta \right|M)$ is the prior probability distribution of the parameters with respect to the model and $P(\left.Y\right|\theta ,M)$ is the likelihood distribution function. $P(\left.Y\right|M)$ is the marginal likelihood or evidence, ensuring that the posterior is properly normalized.

The above expression can also be described as

Posterior distribution ∝ Prior distribution × Likelihood distribution(2)

**Figure 1:**
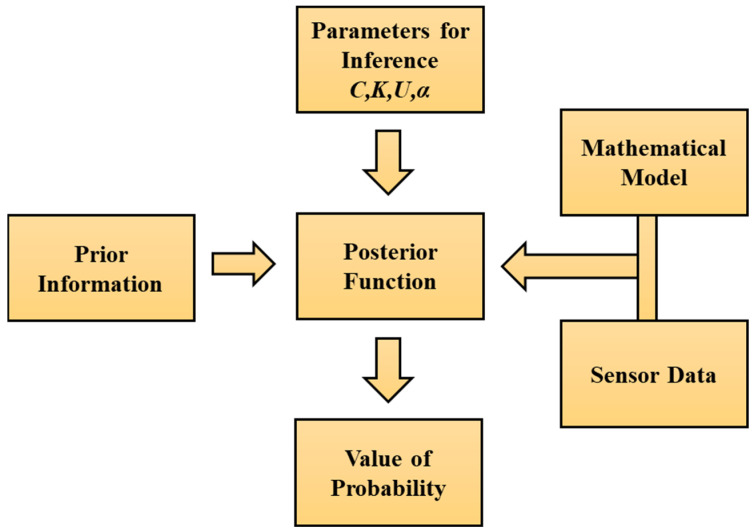
Methodology framework of Bayesian inference for parameter estimation.

The general methodology involves defining uncertainty PDFs for uncertain model parameters, collecting known observed data, and calibrating prior parameters’ PDFs based on observed data by using Bayes’ theorem iteratively until it converges to acceptable levels. Prior distributions can either be informative, e.g., gamma, triangular, etc., or completely non-informative, like uniform distribution of data. Figure 1 illustrates the general process of utilizing Bayesian inference for the determination of parameters, such as bearing parameters (*C*, *K*), unbalance parameters (*U*, *α*), etc., in a rotary system using a mathematical-based model and sensor data. The evaluated posterior function gives information about the model parameter distribution.

The procedure involves iterative refinement rather than a single computation. As new data are acquired, the posterior distribution from each iteration becomes the prior distribution for the next, allowing the cycle to continue. This iterative approach enables the model to continuously adjust, enhancing its accuracy and reliability with each subsequent update.

From Equation (1), it is evident that we must compute the probability $P(\left.Y\right|M)$. To achieve this, we must assess the following integral, which integrates across the whole range of θ parameters:

(3) $$P(Y\;|\;M)=\int_{\theta }P(Y,\theta )d\theta$$

The main issue is that we often cannot assess this integral analytically, necessitating the use of a numerical approximation technique. Computational approaches for sampling such as Markov Chain Monte Carlo (MCMC) are used to evaluate the integral, particularly in complex systems where integral solutions are not feasible. These techniques enable the extraction of samples from intricate posterior distributions. The Metropolis algorithm, which is a variant of the MCMC method, is frequently employed because of its versatility and efficacy. The process entails producing a series of sample values in such a manner that, despite the beginning values being distant from the desired distribution, the samples ultimately approach and align with the posterior distribution. This iterative process begins with an initial guess or prior distribution for the model parameters. At each iteration *t* of the MCMC algorithm, a proposal distribution is employed to generate a candidate parameter value θ, often centered around the current parameter values. The proposed parameter value is then evaluated based on its likelihood given the observed data and the prior distribution. If the new parameter has a higher posterior probability than the earlier parameter value, it would be accepted; otherwise, it would be rejected. This results in a sample of parameters that closely signifies the actual values of the parameters.

The Metropolis acceptance probability a, if the PDF of parameter θ is symmetric, can be computed as follows [39]:

(4) $$a=\min(1,\frac{P(\theta^{t})}{P(\theta^{t-1})})$$

**Figure 2:**
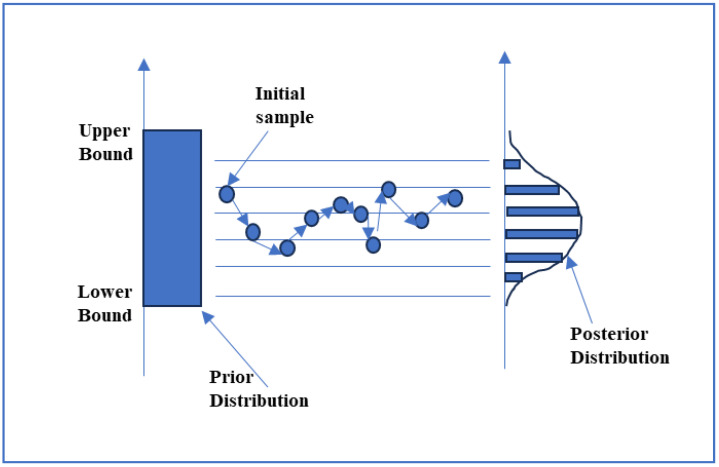
Diagrammatic representation of MCMC-based Metropolis algorithm.

This criterion ensures that the proposed parameter values are accepted with a probability that balances exploration and exploitation, thus guaranteeing that the resulting Markov chain converges to a posterior distribution $P(\left.\theta \right|Y,M)$. The MCMC algorithm iterates through many such proposals, updating the parameter values based on accepted proposals until convergence is achieved. Once convergence is attained, the obtained samples from the posterior distribution are utilized for inference. This includes computing point estimates, such as posterior means or medians, and credible intervals for the parameters of interest. These estimates provide valuable insights into the uncertainty associated with the calibrated parameters. This algorithm runs for a specified number of iterations denoted by *N*, wherein it proposes a new set of parameter values denoted by $\theta^{t}$ at iteration *t* from the proposal distribution. In the simulation loop, it computes the value of likelihood function for parameters $\theta^{t-1}$ at time *t* − 1 and new parameters $\theta^{t}$ at time *t*. Based on Equation (4), it examines the acceptance ratio a. The range of the acceptance ratio varies from 0 to 1 and, depending on the acceptance criteria, some parameter values with a higher acceptance ratio are selected and others are rejected. This process continues iteratively to form a sample of parameters that will represent the posterior distribution, which can then be used to make statistical-based inferences. The Metropolis algorithm is more briefly described in Algorithm 1 and diagrammatically illustrated in Figure 2.

**Algorithm 1:** Metropolis algotrithm**for** i = 1: *N*
    *θ*^*t*−1^ = current parameter    %Propose new parameters    *θ*^*t*^ = proposed parameter    %Calculate likelihoods    Current likelihood = likelihood_function(*θ*^*t*−1^)    New likelihood = likelihood_function(*θ*^*t*^)    %Calculate acceptance ratio    $a=\min(1,\frac{P(\theta^{t})}{P(\theta^{t-1})})$    %Accept or reject proposals    **if** rand < *a* & *a* > 0       *θ*^*t*−1^ = *θ*^*t*^
**end**

## 3. Rotary System Mathematical Model

**Figure 3:**
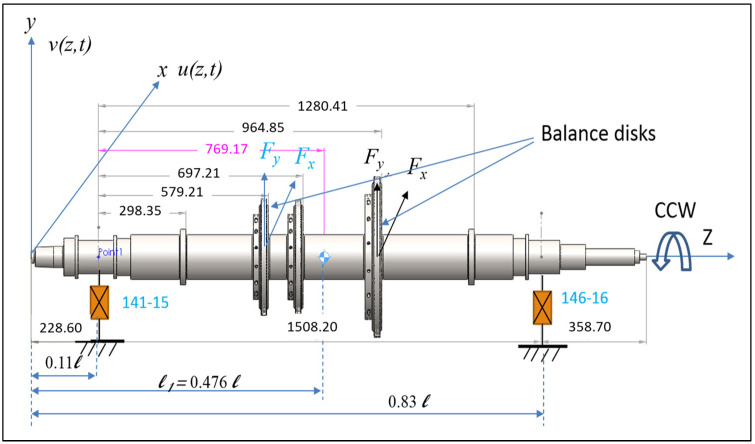
An illustrated turbine rotor–bearing configuration.

To illustrate the current technique, we present a system model consisting of a turbine rotor–bearing, with the specific dimensions shown in Figure 3. This model is derived from an actual turbine rotor found in a local petrochemical facility. It includes a flexible shaft, a three-stage impeller, and two tilting-pad hydrostatic fluid-film bearings with five pads each. The system model is mathematically constructed based on fundamental physical principles. Using the assumed mode method (AMM), equations of motion (EOMs) for the turbine rotor–bearing system were formulated. These equations were subsequently solved to determine the unbalance parameters and to compute unbalance trends using MATLAB 2022 software. The AMM-based model was initially validated against a FEM model and was found suitable for further analysis. Detailed derivations of the mathematical model and additional information on system validation can be found in [20].

After being solved mathematically, the rotor’s unbalanced response in the *x* and *y* directions may be described as u(z,t) and v(z,t), respectively,

(5) $$\begin{array}{cc} u(z,t)\; & =p_{c}^{T}\Phi (z)\cos(\Omega t)+p_{s}^{T}\Phi (z)\sin(\Omega t)\\ & =u_{c}(z)\cos(\Omega t)+u_{s}(z)\sin(\Omega t)=A_{x}\cos(\Omega t-\phi_{x}) \end{array}$$

(6) $$\begin{array}{cc} v(z,t)\; & =q_{c}^{T}\Phi (z)\cos(\Omega t)+q_{s}^{T}\Phi (z)\sin(\Omega t)\\ & =v_{c}(z)\cos(\Omega t)+v_{s}(z)\sin(\Omega t)=A_{y}\cos(\Omega t-\phi_{y}) \end{array}$$

In the above expression, Φ(z) are the flexible modes of a free–free beam, along with two rigid body modes, i.e., translation and rotation. p and q represent the generalized coordinates, with the subscript c and s describing cosine and sine components, respectively. Here, Ω denotes the rotational speed in rad/s.

The measured responses from sensors at two bearing locations are compared with the computed unbalance vibration from the above equations to generate a residual. The model parameters are then derived by minimizing this residual. To obtain orbit data, a pair of orthogonal probes is required at each bearing, typically positioned at 45° to the left and right of the vertical axis. Since unbalance impacts only the vibration components at the rotational speed frequency (1x), the 1x vibration components must be extracted from the overall response for a more detailed analysis. The rotor’s 1x displacement response at the two bearing positions is obtained for further analysis as

(7) $$\left\{\begin{array}{c} u(z_{i},t)=u_{c}(z_{i})\cos(\Omega t)+u_{s}(z_{i})\sin(\Omega t)\\ v(z_{i},t)=v_{c}(z_{i})\cos(\Omega t)+v_{s}(z_{i})\sin(\Omega t) \end{array}\right.\;,\;z_{i}=z_{1}\;\mathrm{or}\;z_{2}\;$$

in which the two distinct bearing points are indicated by the subscripts 1 and 2 at *z*. Consequently, the feature vector with the dimension of 8 × 1 is defined as

(8) $$f={\left\{u_{c1},u_{s1},v_{c1},v_{s1},u_{c2},u_{s2},v_{c2},v_{s2}\right\}}^{T}$$

Residual *e* reflects the total magnitude of the absolute errors between the calculated and actual features, given by

(9) $$e=\sum ∥(f_{T}-f_{M})∥$$

Here, the terms $f_{T}$ and $f_{M}$, respectively, refer to the theoretical (calculated) and measured (actual) feature vectors. The residual is defined as the sum of the absolute differences between the $f_{T}$ and $f_{M}$ vectors. The above residual can be utilized to compute the model error, and a likelihood function can be generated based on it. Thus, the lower the residual error, the higher the parameter acceptance probability.

In rotor dynamics [52], it is established that the dynamic characteristics of a fluid-film bearing can be defined by a stiffness and a damping matrix as

(10) $$k=\left[\begin{array}{cc} k_{xx} & k_{xy}\\ -k_{yx} & k_{yy} \end{array}\right]$$

(11) $$C=\left[\begin{array}{cc} C_{xx} & C_{xy}\\ C_{yx} & C_{yy} \end{array}\right]$$

where $k_{ii}$/$C_{ii}$ and $k_{ij}$/$C_{ij}$ are the direct and cross stiffness/damping coefficients provided by the squeezed film, respectively. According to observation in the above model [20], the cross stiffness and damping of both hydrostatic bearings is negligible in comparison with the direct ones. Consequently, the cross coefficients of stiffness and damping for the bearings are disregarded to streamline the identification process, i.e., *k_ij_* = *C_ij_* = 0.

In an earlier study [20], the process of determining the system’s bearing stiffness and damping coefficients involved minimizing the residual error *e*, using optimization algorithms, such as particle swarm optimization (PSO), during the initial phase of model setup. The resulting bearing constants were then treated as fixed values, and sensor measurements were subsequently used solely to evaluate unbalance parameters in two balance planes during a second phase of PSO. These unbalance parameters are denoted as *U*_1_ and *U*_2_ is the total unbalance acting at bearing locations 1 and 2 along with *α*_1_, *α*_2_ as their corresponding phase angles. Furthermore, the overall static (*U_g_*, *α_g_*) and dynamic (*U_d_*, *α_d_*) unbalances were also calculated.

However, as operational time progressed, a substantial increase in unbalance magnitude was observed after the 120th day, indicating significant changes in the system’s characteristics. A thorough investigation revealed a disruption in the turbine system—specifically, a labyrinth-ring failure—within this period. After replacing the ring, the system was restarted. Follow-up studies noted a marked increase in residual error following this event, suggesting significant inaccuracies in the original model. To address this issue, we refined our model to create a more robust system representation [24]. Specifically, we re-identified the bearing parameters of the system model manually if the residual errors exceeded a certain threshold amount. This adaptive approach led to substantial improvements in the system’s mathematical model.

**Figure 4:**
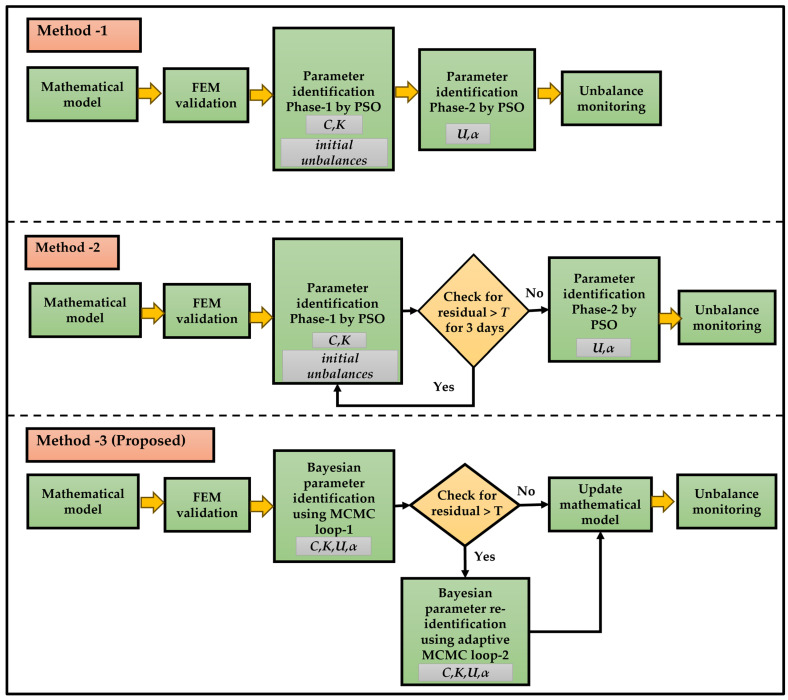
Comparisons of different methodologies for turbine bearing system.

Due to the turbine’s operation under high speeds and loads, the bearings are inevitably affected and will experience variations and alterations over time. Therefore, treating bearing parameters as fixed constants provides an insufficient representation. It is essential to adjust these parameters as time progresses. To address the uncertainties inherent in the complexity of a real-world industrial system and to enhance the accuracy of the system’s mathematical model, we propose a parameter identification method based on Bayesian inference. This method enables continuous updates to the system model, allowing for effective monitoring of unbalance trends over time. The main concept behind the first [20], second [24], and current methods of unbalance monitoring are illustrated in Figure 4 for easier comparison. Further details about our proposed methodology are provided in the following sections.

## 4. Proposed Methodology

### 4.1. Model Updating and Parameter Estimation

The turbine under examination is equipped with two five-pad, tilting-pad, hydrostatic fluid-film bearings. Identifying the characteristics of fluid-film bearings through theoretical calculations presents considerable challenges due to the complex factors influencing their dynamic behavior. In contrast, techniques that use empirical data gathered from actual machinery or test rigs offer a more accurate and practical evaluation of bearing dynamics [19,20,21]. The parameter estimation approach described here follows a Bayesian framework, which explores multiple pathways to deduce the optimal values of key parameters within the context of the rotor system. Detailed information on each step of the proposed methodology is provided below.

Step 1: Assess a prior PDF for the interest parameters.

Bayesian inference incorporates prior information or assumptions about the parameters of interest by using a prior distribution in the analysis. This distribution reflects the uncertainty surrounding the parameters before any data are observed. Various probability density functions (PDFs), such as the normal or uniform distribution, can represent this prior distribution. In our study, the parameters of interest include the damping coefficients (*C_x_*_1_, *C_y_*_1_, *C_x_*_2_, and *C_y_*_2_) and spring constants (*K_x_*_1_, *K_y_*_1_, *K_x_*_2_, and *K_y_*_2_) as well as the initial unbalances (*U*_1_, *α*_1_, *U*_2_, and *α*_2_) at the two different bearing locations. Here, we assume a uniform distribution over a specified range for these parameters, based on prior information or expert knowledge. This distribution captures the range of possible values for each parameter.

Step 2: Compute the likelihood for given set of parameters.

The likelihood function measures the probability of witnessing the data, considering the model and specific parameter values. It quantifies the degree to which the model, using certain parameters, accurately describes the observed data. In our case, the likelihood function is an exponential function depending on the calculated residual error for a set of parameters.

Step 3: Calculate the posterior distribution as a result of the variation in parameters of the model.

Bayesian inference uses observed data to update previous information about the parameters and generate the new posterior distribution. This distribution represents the revised understanding or convictions of the parameters, considering the data that have been observed. The posterior distribution integrates information from both the prior distribution and the likelihood function, resulting in a consistent depiction of uncertainty in the parameters. The computation of posterior distribution can be established by Equation (1).

Step 4: Generate several samples from the posterior distribution using the Metropolis algorithm in MCMC.

The Metropolis algorithm is a Monte Carlo method that utilizes Markov chains to sample from proposal probability distributions and evaluate the posterior distribution using Bayesian inference. The process involves generating a series of samples and evaluating the posterior distribution by repeatedly suggesting new parameter values from the proposal distribution and determining whether to accept or reject them based on an acceptance criterion evaluated by Equation (4). This approach enables an efficient investigation of the parameter space and estimation of the posterior distribution.

Step 5: Compute the parameters of interest from the obtained sample by an algorithm and determine the mean or median for the parameters of interest.

After collecting an adequate number of samples from the posterior distribution, it is possible to compute summary statistics, such as credible intervals, mean, or median, to better understand the uncertainty in the parameter estimations. Credible intervals offer a range of believable values for the parameters, whereas the mean or median show the center tendency of the distribution or more optimal or average solutions. We have utilized the mean values of the parameters of interest as the parameter set of a particular day for further calculations based on that.

Step 6: Assess the model outputs using new identified parameters, verify the accuracy of the resultant residual error curve, and compare it to the previous methods.

**Figure 5:**
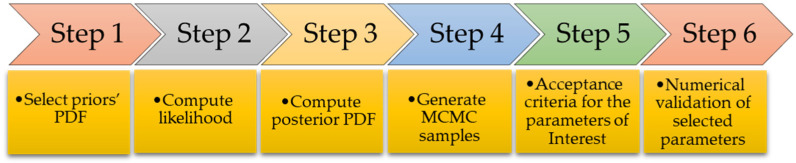
Steps for Bayesian inference using MCMC.

Ultimately, the model is assessed by employing the estimated parameters and its performance is evaluated depending on how the overall residual error varies compared to the errors in previous methodologies. The aim at every iteration is to keep the residual error values less than a threshold value *T*, so that the model does not change much from the real system. This allows for iterative modification of the model. Figure 5 shows in an illustrative method for the above-mentioned steps.

**Figure 6:**
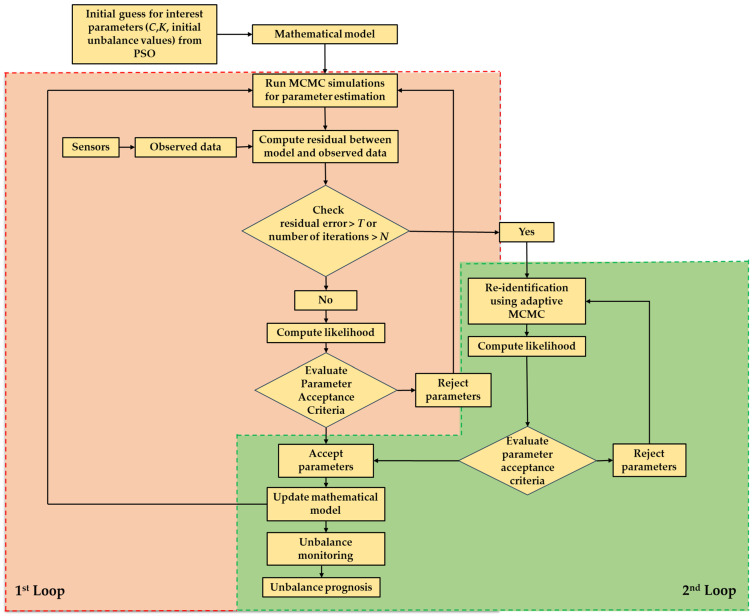
Flowchart for the proposed methodology with dual MCMC loops.

Thus, this methodical approach to evaluate the parameters within a Bayesian framework offers a rigorous methodology for assessing and updating a rotor–bearing model. It considers uncertainty and updates knowledge as new data are obtained. The above methodology is further improved to take into consideration the case of irregularity in the system, such as a sudden change in the system, which can lead to a substantial change in parameter values. For this, one extra re-identification MCMC loop has been added for better exploration of the parameter space. The detailed process of the proposed methodology is depicted in Figure 6.

From Figure 6, we can see that a dual-loop MCMC approach was utilized for the parameter identification procedure, as described more in detail below. First Loop or Main MCMC loop: For each individual data point collected under different conditions or at different times, the process of estimating parameters includes following a set of organized steps inside a Bayesian framework, utilizing the Metropolis algorithm. Initially, a fresh set of parameter values is suggested by randomly selecting from a uniform distribution centered on the existing parameters. Next, the model response is computed using interest parameters, which are initially specified by guess values and later by the mean of the accepted samples in each loop, and the disparity between this model response output and the observed data response is assessed. The likelihoods of the proposed parameters are calculated based on the normal distribution errors and subsequently the acceptance ratio is computed, which is the proportion of the posterior probability of the proposed parameter set to the current set of data of interest, considering the computed likelihoods and prior distribution PDF. The accepted parameter set is evaluated using the Metropolis criterion, where a random number from the uniform distribution of [0, 1] is compared against the acceptance ratio. If the random number is lower than the acceptance ratio, the proposed set is accepted and replaces the present one. Otherwise, it is rejected. This approach favors parameters with higher acceptance ratios, making them more likely to be accepted than those with lower ratios. This process is repeated multiple times to construct a posterior distribution that accurately represents the parameter space of interest.

Second Loop or Adaptive Re-identification MCMC loop: The re-identification MCMC loop is a sophisticated component of the proposed MCMC architecture. It is triggered when the errors linked to the current parameter set are above a predetermined threshold value *T*. This loop is especially tailored to enhance the parameter estimations under difficult settings where the primary MCMC loop fails to yield satisfying outcomes. During the re-identification loop, a random walk is used to suggest a new set of parameters. The step size of the random walk is changed based on the acceptance rate of previous loop values. For every new set of parameters of interest, the model generates an output response, and the error is computed by comparing this output with the observed set of data. The likelihood function is computed by considering it to be an exponential function of error. Subsequently, an acceptance ratio is computed by considering the likelihood and prior distribution PDF of parameters. The new parameter set is determined to be either accepted or rejected based on the acceptance ratio, using the Metropolis criteria. If the new parameters are approved, they will modify the existing set. This loop iterates until the parameters produce an error that is lower than the specified threshold *T*. The accepted parameters through this process are sent back to the main MCMC loop for the next day.

The primary purpose of the main MCMC loop is to effectively explore the parameter space and obtain samples from the posterior distribution on a normal operation. On the other hand, the re-identification MCMC loop serves as a supplementary mechanism to avoid being stuck in local optima or areas of high error in the parameter space during an unexpected event occurrence. The approach tries to provide robust and accurate parameter estimation by integrating these two Markov Chain Monte Carlo (MCMC) processes. This integration takes into consideration the uncertainties present in both the model and observed data.

### 4.2. Methodology for Prognosis

The concept of unbalance prognosis facilitates comprehensive knowledge of the evolution of unbalance, its anticipated behavior, and the prompt execution of appropriate maintenance interventions. The process involves data collection, continuous monitoring of unbalanced faults, model training, and evaluation of prediction accuracy related to unbalanced faults over a designated period. This approach employs established forecasting techniques, renowned for elucidating the temporal patterns of the time-series curve in long-term evaluations. Therefore, the steps for prognosis can be established as the following:

- Data acquisition—1x vibration data are obtained from the sensors located at the two bearing locations.
- Proposed model-based unbalance computations—the overall unbalance fault values *U_g_*, *U_d_*, *α_g_*, and *α_d_* from the proposed methodology are evaluated.
- Unbalance monitoring—monitoring of the unbalance faults for the presence of trends over a span of time.
- Unbalance prognosis—this involves the forecasting of the evaluated unbalance faults to understand their future trends and better predict the nature of their occurrence.

**Figure 7:**
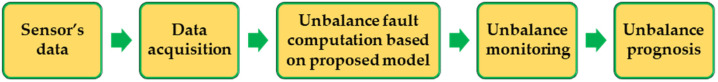
Steps for prognosis.

Figure 7 illustrates the aforementioned stages in a schematic manner, depicting their sequential occurrence.

In our methodology, we have considered the LSTM method to be more appropriate for long-term forecasts for the prognosis task, as discovered from our previous work [24]. LSTM is an enhanced variant of recurrent neural network (RNNs) that mitigates the vanishing gradient problem often seen with RNNs. These methods are mostly used in problems related to sequence predictions, including time-series forecasting, natural language processing, and voice recognition. This technique was chosen for its ability to reliably forecast the distributions of non-linear regression data over an extended duration. This method incorporates memory cells and three specific gates: the input gate, forget gate, and output gate, which allow the neural network to efficiently retain, eliminate, and apply knowledge across sequential inputs. This enables the method to accurately capture both transient and enduring links within the data.

**Figure 8:**
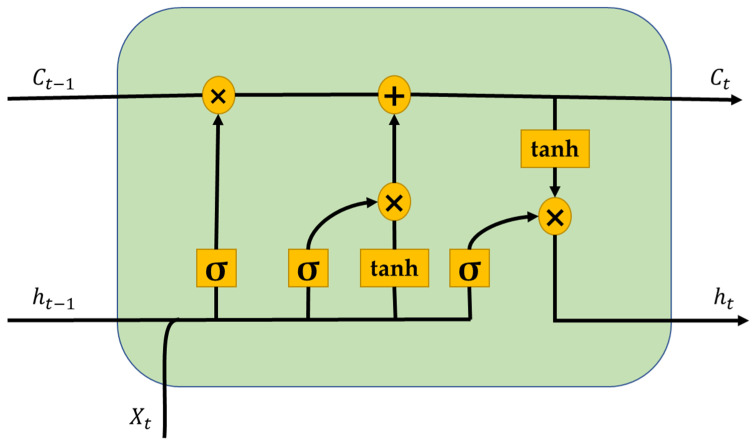
Diagrammatic representation of LSTM unit cell.

For a time series network at time *t*, if $x_{t}$ is the input data for the LSTM cell, $h_{t-1}$ is the output of the previous cell, $c_{t}$ is the memory value of the current memory cell, $c_{t-1}$ is the memory value of the previous memory cell, and $o_{t}$ is the output of the LSTM cell, then the calculations for LSTM outputs within a cell unit are given by following equations:

(12) $$c_{t}=\tanh(w_{c}[h_{t-1},x_{t}]+b_{c})$$

(13) $$i_{t}=\sigma (w_{i}[h_{t-1},x_{t}]+b_{i})$$

(14) $$f_{t}=\sigma (w_{f}[h_{t-1},x_{t}]+b_{f})$$

(15) $$c_{t}=f_{t}*c_{t-1}+i_{t}*c_{t}$$

(16) $$o_{t}=\sigma (w_{o}[h_{t-1},x_{t}]+b_{o})$$

(17) $$h_{t}=o_{t}*\tanh(c_{t})$$

where $w_{i}$, $w_{c}$, $w_{f}$, and $w_{o}$ are the weights and $i_{t}$, $f_{t}$, and $o_{t}$ represent the input gate, forget gate, and output gate, respectively. tanh and σ (sigmoid) are the two activation functions used for calculations in a simple LSTM cell. Figure 8 represents a unit cell of LSTM with elements described above.

## 5. Results and Discussion

As can be seen from Figure 6, sensor data from the bearing positions are extracted as observed data. As per the proposed methodology, the first step entails the configuration of the eight initial bearing parameters (*C_x_*_1_, *C_y_*_1_, *C_x_*_2_, *C_y_*_2_, *K_x_*_1_, *K_y_*_1_, *K_x_*_2_, and *K_y_*_2_) as well as the initial unbalances (*U*_1_, *U*_2_, *α*_1_, and *α*_2_) at the two bearing locations. This is to determine initial values for the parameters by utilizing prior assessments, which involves using past knowledge. According to the evaluations in [20], we set the initial values of the bearing parameters as the ones initially determined by the PSO in our previous method as a starting point or initial guess of the bearing parameters.

After setting the initial parameters, the Bayesian framework enables the estimation of unknown parameters by updating their probability distribution PDFs with observed data. These are essential for precisely simulating the dynamic behavior of the rotor system. In order to streamline the parameter estimation process, it is assumed that the values of the stiffness and damping parameters only fluctuate within specific ranges, which are given as uniform PDF ranges. The specified ranges establish the potential values that the parameters can assume, considering the physical limitations and attributes of the system being studied.

The range of uniform PDFs offers a direct and pragmatic method for expressing uncertainty regarding the parameters. This allows for a comprehensive examination of potential parameter values during the MCMC sampling process. The Bayesian framework enables the exploration of the parameter space by setting these ranges to locate high-probability regions that align with the observed data. The specified range values for the stiffness and damping parameters act as limits within which the MCMC algorithm repeatedly selects potential values for these parameters. During each iteration of the MCMC loop, parameter values are stochastically sampled from the defined ranges. Subsequently, the probabilities of these values are assessed by considering all the observed data and the various parameters values in the mathematical model. At each iteration of the simulation, the residual error from Equation (9) is calculated by comparing the outputs of the mathematical model with the observed sensor data. The residual error is a crucial measure of the precision of the existing model parameters.

The method iteratively checks if the residual error exceeds a predetermined threshold, T (a 30% increase in the residual value), or if the number of iterations reaches a specified limit, N (set to 200). If either condition is met, the process shifts to an adaptive MCMC technique to further refine the model parameters. The adaptive re-identification MCMC methodology adjusts the proposal distribution to more effectively explore the parameter space, using a random walk MCMC approach to identify the model parameters.

The step-size for random walk computation is adaptively calculated based on the acceptance ratio of the algorithm. A larger step size aims to rigorously explore the parameter space, while a smaller step size helps to refine the search around promising regions. This adaptive search helps to strike a balance between exploration and exploitation for effective identification of parameters. By repeatedly going through the loop, the method reaches a point when it converges to the posterior distribution of the parameters. This distribution reflects the updated probability distribution based on the observed data. The posterior distribution found gives vital information on the uncertainty related to the parameters. This allows for a more accurate and resilient assessment of the bearing characteristics in the rotor system, which is crucial for problem diagnosis and prognosis. After accepting a set of parameters, the mathematical model is revised to accurately describe the dynamics of the system. This revised model is now more closely matched with the actual behavior of the rotor system. The updated model is used to continuously monitor the system’s unbalance.

### 5.1. Unbalance Monitoring

**Figure 9:**
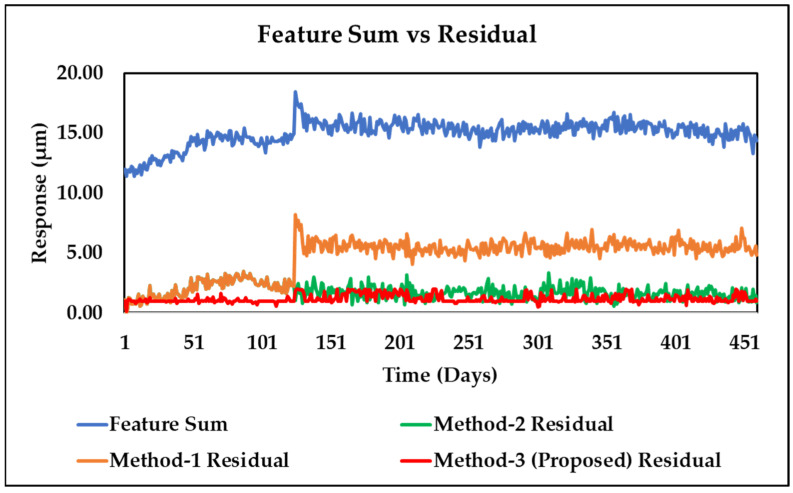
Plot for the feature sum and residual comparing the old and new approaches.

**Figure 10:**
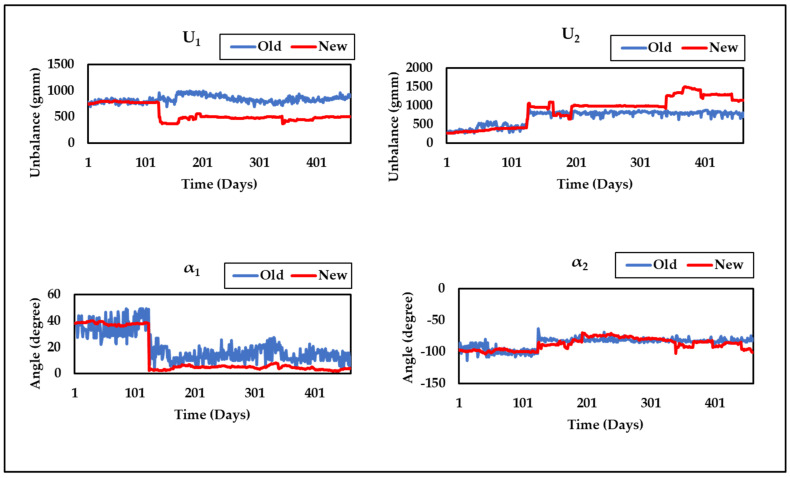
Curves of *U*_1_, *U*_2_, *α*_1_, and *α*_2_ comparing the old and new approaches.

**Figure 11:**
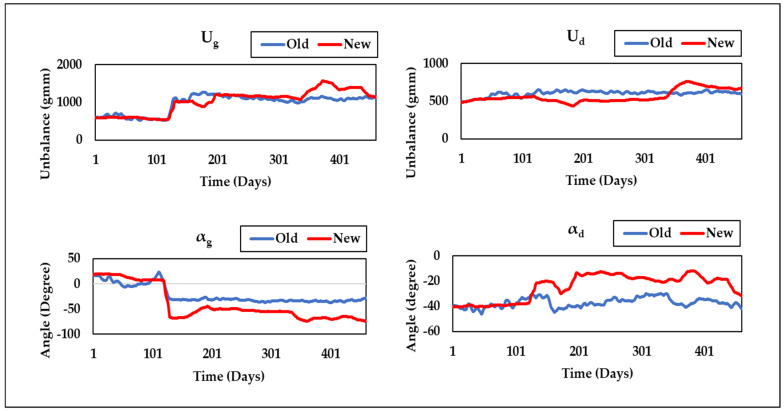
Curves of *U_g_*, *U_d_*, *α_g_*, and *α_d_* comparing the old and new approaches.

Figure 9 shows that the proposed technique (Method-3) consistently maintains the lowest residual throughout the run, indicating improved performance over previous methods. The unbalance parameter curves obtained by Method-3 (New) at the two bearing positions (*U*_1_, *U*_2_, *α*_1_, and *α*_2_) and the overall static and dynamic unbalance (*U_g_*, *U_d_*, *α_g_*, and *α_d_*) of the system are displayed in Figure 10 and Figure 11, alongside results from Method-1 (Old). These curves provide a clear visual representation of the unbalance behavior and its evolution over time, offering a more comprehensive view of the rotor system’s dynamics. Figure 10 and Figure 11 also reveal a sudden change in unbalances around the 120th day, which is attributed to the replacement of labyrinth rings that altered the unbalance state.

**Figure 12:**
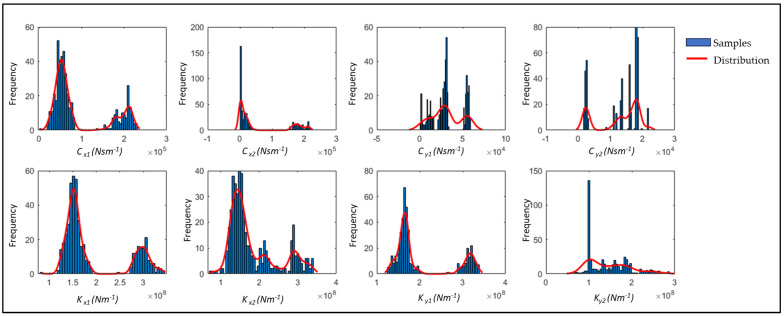
Overall distribution of bearing parameters.

Figure 12 shows the overall histogram distribution of the estimated parameters, highlighting the level of uncertainty associated with each one. This information is essential for assessing the reliability and stability of parameter estimates, providing insights into their underlying distributions, which are valuable for maintenance and fault diagnosis decisions. The figure reveals a pattern of dual peaks in most parameter values, suggesting a significant shift in the system state and a corresponding increase in parameter variation. This observation aligns with a real event during which a labyrinth ring broke, altering the system’s behavior and causing shifts in bearing parameters. If the old parameters are used post-incident, the residual error remains elevated; however, the proposed methodology allows for ongoing parameter adjustment to better understand and respond to such changes.

**Figure 13:**
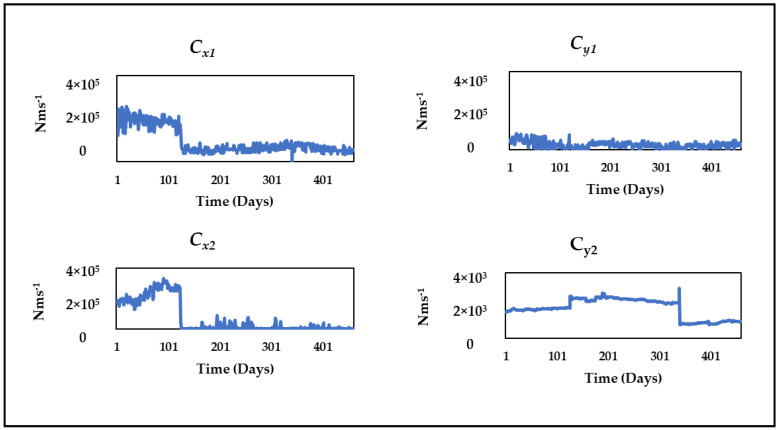
Variation in mean damping parameters *Cs*.

**Figure 14:**
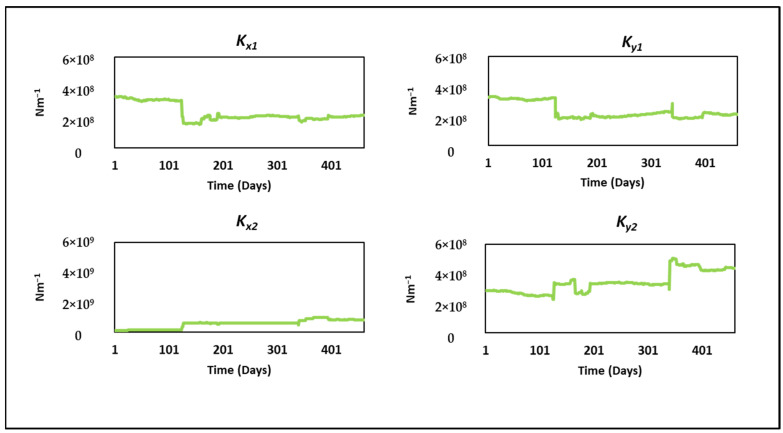
Variation in mean stiffness parameters *Ks*.

Figure 13 and Figure 14 illustrate the evolution of the mean damping and stiffness parameters, respectively, over the operational period. These charts demonstrate the convergence of parameter estimates and offer insights into parameter variation over time.

Table 1 displays a juxtaposition of the residual errors acquired from the suggested model with the previous techniques that we established using the deterministic parameter identification method in [20], also denoted by Method-1, as well as the adaptive methodology in [24], denoted by Method-2. The results unequivocally demonstrate that the proposed MCMC-based model updating technique denoted by Method-3 outperforms both Method-1 and Method-2 in terms of both the sum and the average of the residuals. The overall percentage residual error reduction of our proposed Method-3 is as high as 74.48% compared to Method-1. In contrast, about 62.2% reduction was found in the previous Method-2. The substantial decrease in residual errors demonstrates the efficacy of the proposed method in capturing uncertainty and enhancing the precision of parameter estimations.

The use of Bayesian inference and MCMC sampling in the proposed methodology offers several advantages over traditional deterministic approaches. By incorporating parameter uncertainty and dynamically updating probability distributions based on observed data, this approach provides a more comprehensive and reliable assessment of bearing characteristics. This, in turn, enhances the ability to detect and predict unbalance faults, enabling more effective maintenance planning and decision-making.

Moreover, the proposed methodology highlights the potential for further improvements through advanced MCMC sampling techniques. These techniques enable more efficient exploration of the parameter space, resulting in more precise parameter estimations and enhanced model performance. In summary, the proposed model presents a robust approach for fault monitoring in rotor systems, allowing for more accurate detection of unbalance faults. Additionally, it supports maintenance decisions by offering valuable insights into the behavior and condition of turbine rotor systems, ultimately increasing system reliability and operational efficiency.

### 5.2. Unbalance Prognosis

After confirming the alignment between the physical model and the actual system, the model may be used for unbalance prediction, facilitating a more precise evaluation of potential unbalance. This not only improves the system’s overall dependability but also facilitates its safe and timely maintenance. In this situation, forecasting functions is an essential tool for anticipating the future behavior of the system. This work has utilized LSTM models for monitoring and prognostic analysis. The LSTM model has superior performance with extensive historical data and can be inherently more complicated as it requires meticulous tweaking of hyperparameters to provide precise outcomes; yet, it is better suited for long-term forecasting.

**Figure 15:**
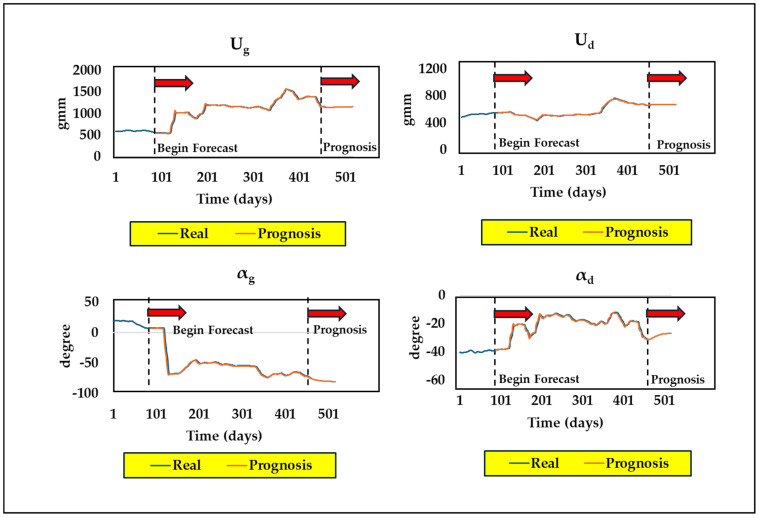
Prognosis of unbalance fault parameters of turbine rotor–bearing system.

The unbalance dataset fed into the forecasting model was first smoothed using the “smooth” function present in the MATLAB Curve Fitting Toolbox using the “rlowess” method, a robust linear regression function defined over a specified window length. This pre-processing step was also performed to remove noise from the data. The prognostic algorithm was developed with the MATLAB 2022 software for predictions. The online execution for daily predictions adheres to a defined approach. The model is first trained on data from the first 90 days. Then, each subsequent day is forecasted using the model. This procedure is then repeated every day, with the data from each new day included in the training dataset via an expanding window methodology. The rationale for using expanding windows instead of sliding windows is because expanding windows seem to provide better predictions as per our previous observations. This facilitates the ongoing enhancement of the model. Our forecasting model was used to predict the next 60-day period, from a total dataset of 460 days. Figure 15 shows the overall illustration of real occurrences with the prognosis of unbalance parameters over the days of operation.

Furthermore, the LSTM model was tested for different hidden units; finally, with 15 hidden units, a dropout rate of 0.2 was implemented, as it seemed to give lower values of root mean square error (RMSE). The model tackles the forecasting issue by optimizing according to the “best validation loss” criteria and uses the “adam” optimizer function. The LSTM model then undergoes training of over 200 epochs and a ‘Mini Batch Size’ of 16 was selected with a learning rate of 0.01. The anticipated curve closely matches the actual one, as can be seen from Figure 15, indicating its reliability for identifying unbalance faults in the long run.

## 6. Conclusions and Future Work

### 6.1. Conclusions

This research focuses on a critical aspect of engineering systems, particularly rotor-based structures: uncertainty in parameter estimation. Models are essential for computations and analysis in engineering, yet they inherently contain uncertainties that can impact their accuracy. Addressing these uncertainties is crucial, as precise parameter estimates are vital for detecting defects in turbine rotor systems. Conventional deterministic approaches often overlook these uncertainties, attributing them to inherent model flaws.

To address this challenge and improve the precision of our mathematical model for rotor system unbalance monitoring, this work applies Bayesian inference and MCMC sampling techniques to enhance parameter evaluation. This approach’s effectiveness is demonstrated in an industrial turbine rotor–bearing system in this research, showing a significant reduction in model error—up to 74.48%—through continuous model updating. The demonstration also proves Bayesian and MCMC methods work well for this type of model uncertainty.

Additionally, this research highlights enhanced parameter searching through sophisticated MCMC sampling techniques, such as dual-loop MCMC, which combines a regular MCMC loop with an adaptive re-identification MCMC loop. The adaptive loop activates when conventional MCMC approaches fail to sufficiently reduce error, introducing adaptive proposals to refine the proposal distribution, thereby improving parameter estimation under challenging conditions. These advanced sampling strategies enable more efficient exploration of the parameter space, resulting in greater estimation accuracy and improved model performance.

With accurate parameter estimates in place, the model supports real-time unbalance fault monitoring, enabling trend extrapolation and future forecasting using an LSTM-based method. The LSTM model’s hyper-parameters are carefully tuned to minimize the RMSE, making unbalance fault forecasts useful for predictive maintenance and strategic decision-making to optimize system performance and reduce downtime.

In summary, the model developed in this research provides a powerful tool for monitoring and predicting unbalance faults in rotor systems. It enhances maintenance strategies by offering critical insights into bearing and turbine rotor system behavior and condition, facilitating accurate detection in complex, real-world scenarios. Ultimately, this approach promises increased reliability and operational efficiency, showcasing the model’s effectiveness in managing inherent uncertainties in engineering calculations.

### 6.2. Future Work

Future research may investigate the integration of more sophisticated sampling techniques to improve the accuracy of parameter estimation. Furthermore, incorporating advanced machine learning models or hybrid methodologies that merge physics-based and data-driven techniques may enhance the system’s predictive capabilities. Extending the model’s application to various rotor systems or wider engineering contexts could illustrate its generalizability and robustness. These advancements offer potential for enhancing uncertainty management and decision-making in engineering computations.

## Figures and Tables

**Table 1:** Comparison of residuals from the proposed and the previous methods.

Methods for Model Updating	Residual Sum	Average Residual	Residual Reduction (%) Compared to Method-1
Method-1	2114.87	4.60	_
Method-2	799.30	1.74	62.20
Method-3	539.59	1.17	74.48

## Data Availability

The data presented in this study is available on request from the corresponding author due to privacy.
